# Behavior of Microbubbles on Air–Aqueous Interfaces

**DOI:** 10.1021/acs.langmuir.4c02546

**Published:** 2024-10-25

**Authors:** Hyunhong J Min, Luca Bau, Stephen J Payne, Eleanor P Stride

**Affiliations:** 1 Institute of Biomedical Engineering, Department of Engineering Science, University of Oxford, Oxford OX3 7LD, U.K.; 2 Institute of Applied Mechanics, National Taiwan University, Taipei 10617, Taiwan

## Abstract

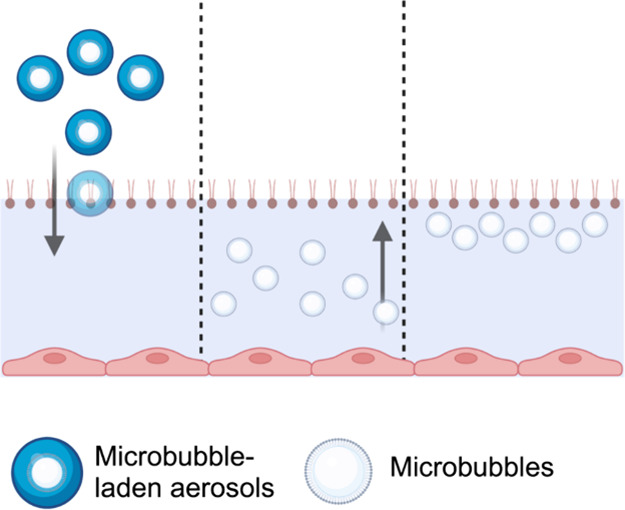

Animal-derived lung surfactants have saved millions of lives of preterm neonates with neonatal Respiratory Distress Syndrome (nRDS). However, a replacement for animal-derived lung surfactants has been sought for decades due to its high manufacturing cost, inaccessibility in low-income countries, and failure to show efficacy when nebulized. This study investigated the use of lipid-coated microbubbles as potential replacements for exogenous lung surfactants. Three different formulations of microbubbles (DPPC with/out PEG40-stearate and poractant alfa) were prepared, and their equilibrium and dynamic surface tensions were tested on a clean air–saline interface or a simulated air-lung fluid interface using a Langmuir–Blodgett trough. In dynamic surface measurements, microbubbles reduced the minimum surface tension compared with the equivalent composition lipid suspension: e.g., PEG-free microbubbles had a minimum surface tension of 4.3 mN/m while the corresponding lipid suspension and poractant alfa had 20.4 (*p* ≤ 0.0001) and 21.8 mN/m (*p* ≤ 0.0001), respectively. Two potential mechanisms for the reduction of surface tension were found: Fragmentation of the foams created by microbubble coalescence; and clustering of microbubbles in the aqueous subphase disrupting the interfacial phospholipid monolayer. The predominant mechanism appears to depend on the formulation and/or the environment. The use of microbubbles as a replacement for exogenous lung surfactant products thus shows promise and further work is needed to evaluate efficacy in vivo.

## Introduction

11% of neonates are born before 37 weeks of gestation, resulting in an estimated 13–15 million premature babies per year globally. Preterm birth, despite improvements in management, is the leading cause of infant morbidity and mortality, and neonatal respiratory distress syndrome (nRDS) remains one of the main challenges in neonatology.

Neonates with nRDS are characterized by poor lung function, such as low functional residual capacity (FRC) and low lung compliance.^1^ Poor lung function creates poorly ventilated lung regions and subsequently activates a compensatory mechanism to shunt blood perfusion from poorly ventilated regions of the lungs to well-ventilated regions. In nRDS, however, vasoconstriction increases the risk of cerebral and pulmonary hemorrhage and ventilation-perfusion mismatch due to increased blood pressure in the upstream pulmonary arteries.^2^ The ventilation–perfusion mismatch increases the risk of hypoxemia and may cause developmental disabilities, such as cerebral palsy.

High surface tension in the air–aqueous interface in alveoli and small airways is the leading factor causing nRDS.^3^ High surface tension in these regions means that higher air pressure is required to increase lung volume. High air pressure, in turn, increases the recoil pressure of airways and increases the risk of airway collapse upon exhalation. A histological study in surfactant-deficient rabbits has shown that the amount of damage is related to the magnitude of the changes in airway pressure.^4^ Poorly supported respiratory bronchioles and alveolar ducts in preterm neonates are particularly vulnerable to collapse.^5^ Higher pressures can also cause overdistension of well-ventilated regions of the lungs, which further decreases lung compliance. Furthermore, airway closures can cause infiltration of proteins and/or a buildup of fluid, resulting in low functional residual capacity.

Less-invasive surfactant administration (LISA) is increasingly becoming the main standard of treatment for the early onset of nRDS.^6^ LISA was developed to avoid the use of mechanical ventilation and administer lung surfactant while preterm neonates are spontaneously breathing. It involves laryngoscopy and the introduction of a thin, soft catheter into the larynx and the trachea. LISA has been shown to reduce the need for mechanical ventilation, but it is a high-risk procedure requiring neonatologists experienced in airway management and still carries a high rate of side effects and complications.^7^ Without correct manipulation, it leads to complications, such as surfactant reflux, acute desaturations, uneven delivery of surfactant, and pneumothorax, eventually leading to the need for mechanical ventilation.

Inhalation of lung surfactant at birth could hypothetically circumvent the main complications of intratracheal surfactant administration such as uneven distribution of lung surfactants and surfactant reflux. It can potentially deliver surfactants directly to the respiratory bronchioles and alveolar ducts, improving short and long-term outcomes of preterm babies.^8^ Conventionally nebulized surfactants fail to reach the lungs and deposit on the narrow airways in the extrathoracic region.^9,10^ In a recently terminated Phase II study (NCT03235986), nebulized poractant alfa failed to show efficacy.^11^ This was attributed to an insufficient amount of surfactant reaching the lungs. Poor lung delivery appears to be an inherent drug delivery barrier in preterm neonates because of narrow extrathoracic airways and the high flow rate required to achieve oxygenation (approximately at 8 L/min).^12^

Microbubbles, consisting of a gas core surrounded by a surfactant or lipid coating may offer a potential solution to this problem. Our previous study found that microbubble-containing aerosols improve lung penetration in a 3D-printed preterm neonate model.^13^ In addition to improved lung penetration, microbubbles may be effective in reducing the surface tension at the air–aqueous interface. The ability of microbubbles to transfer lipids to other lipid membranes (e.g., cellular lipid bilayer and artificial lipid layers) has previously been reported^14,15^ but has yet to be studied on air–aqueous interfaces. The exact mechanism of the transfer is unknown, but the published results suggest that microbubbles may have the ability to improve the adsorption of phospholipids on the air–aqueous interface and thus achieve low surface tension even with small amounts of phospholipids.

**Figure 1:**
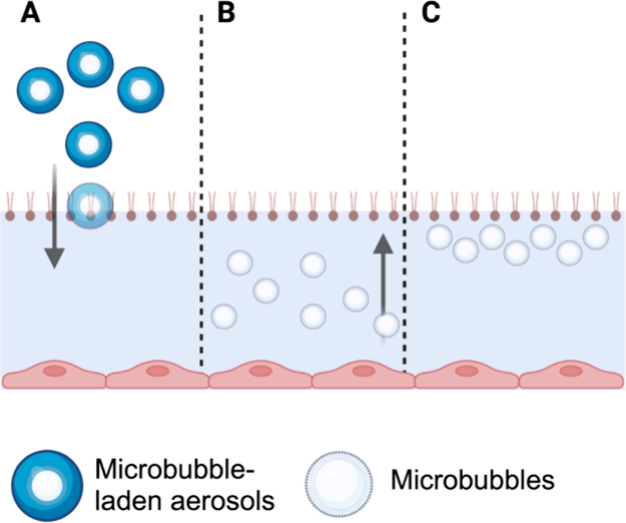
Schematic diagram depicting the hypothesized process by which microbubbles interact with the phospholipid monolayer at the air–aqueous interface. (A) Microbubble-laden aerosols deposit in the lung lining fluid by sedimentation. Microbubbles float to the air–aqueous interface of deposited droplets (B), resulting in clusters that may reduce the surface tension (C). The diagram is not drawn to scale.

In this study, we hypothesized that incorporating microbubbles into aerosols used for nRDS treatment could improve phospholipid deposition as follows: first, aerosol droplets with aerodynamic diameters below 2 μm^10^ will deposit onto the lower side of the airways by sedimentation due to gravity (Figure 1A). Microbubbles, which have a lower density than water, will float to the upper air–aqueous interface of the deposited droplets (Figure 1B). This subsequently results in clusters of microbubbles resting near the phospholipid monolayer at the air–aqueous interface (Figure 1C). The inherent instability of microbubbles was speculated to facilitate better transfer of phospholipids and reduction of air–aqueous surface tension. To test this hypothesis, the effect of phospholipid-coated microbubbles on the surface tension of air–aqueous interfaces was studied.

## Methods and Materials

### Materials

Dipalmitoylphosphatidylcholine (DPPC), 1,2-dipalmitoyl-*sn*-glycero-3-phosphoglycerol, and sodium salt (DPPG) were purchased from Avanti Polar Lipids, Inc. (Alabaster, AL, USA) as a 25 mg/mL solution in chloroform. Polyoxyethylene (40) stearate (PEG-40S), sodium chloride, palmitic acid, propylene glycol, and chloroform were purchased from Sigma-Aldrich Company Ltd. (Dorset, UK) as a powder. Poractant alfa (Curosurf) was purchased from Chiesi Limited, Manchester, UK. An 8 mL glass vial was purchased from VWR. Perfluorobutane was purchased from BOC Group plc, Woking, UK.

### Microbubble Preparations

**Figure 2:**
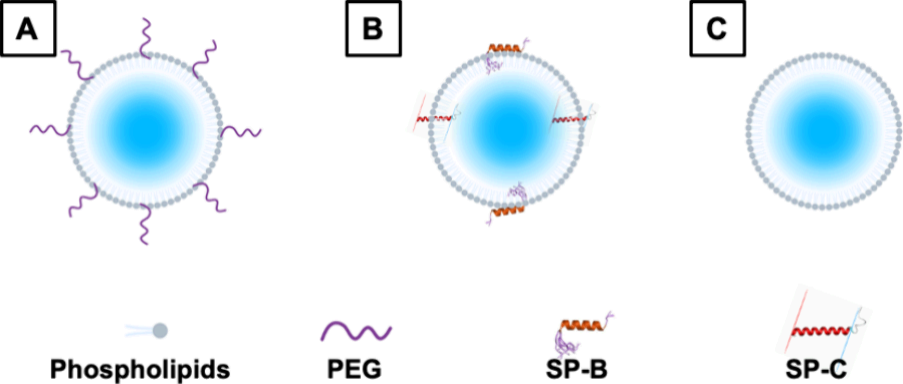
Three different types of microbubbles were produced. The first on the left (A) is a PEGylated microbubble consisting of phospholipids, PEGylated lipids, and a gas core. In the middle (B) is a microbubble composed of poractant alfa, which is expected to contain pulmonary surfactant proteins B and C (SP-B and SP-C, respectively). Lastly, panel (C) represents a PEG-free microbubble composed of phospholipids, lipids, and a gas core.

Three different types of microbubbles were produced (Figure 2). The first microbubble, shown in Figure 2A, was formulated with PEGylated lipid (i.e., PEG40-S) and phospholipid (i.e., DPPC), and this is termed PEGylated microbubbles. PEGylated microbubbles were formulated and prepared based on previous literature^16^ and were known to produce stable microbubbles. In a feasibility study of a clean air–aqueous interface, PEGylated microbubbles were employed to investigate their influence on the surface tension of the interface.

Poractant alfa, which failed to show clinical efficacy at low concentrations,^11^ was formulated into microbubbles (Figure 2B). Poractant alfa microbubbles were tested on a clean air–aqueous interface to examine whether the surface-tension-lowering ability of poractant alfa could be enhanced by formulating it in microbubbles. Lastly, a microbubble formulation without PEGylated lipids was made and is termed PEG-free microbubbles. PEG-free microbubbles were formulated to avoid potential hypersensitivity to PEG^17^ and the use of excipients that are not naturally present in preterm neonates. PEG-free microbubbles were tested for their surface activity in simulated lung conditions.

The PEGylated microbubble was composed of DPPC (Avanti Polar Lipids, USA) and PEG40-S (Sigma-Aldrich, UK), dissolved in chloroform (Sigma-Aldrich, UK). They were mixed in an 8 mL glass vial at a molar ratio of 9:1 (DPPC:PEG40-S). Six mg of DPPC (or 240 μL of 25 mg/mL) and 1.86 mg of PEG40-S (or 74.4 μL of 25 mg/mL) were measured in the vial. The mixture was subsequently dried using a vacuum evaporator at 30 °C for 3 h to allow for chloroform to evaporate. The obtained dry lipid film was suspended in 3 mL of 0.9% w/v NaCl aqueous solution for 1 h in a temperature-controlled shaker at 60 °C and 800 rpm. Lipids were then homogeneously dispersed for 60 s using a sonicator (Q125, QSonica, Newtown, CT, USA) at a power setting of 50% with the tip completely immersed in the lipid solution. This was the DPPC/PEG40-S lipid suspension.

The DPPC/PEG40-S lipid suspension was subsequently used to make microbubbles. The sonicator tip was placed at the air–aqueous interface under constant perfluorobutane (PFB) flow (The BOC Group plc, UK) and sonicated for 30 s at a power setting of 90%. Immediately after production, the vial containing the MB suspension was capped and placed in ice.

For the washed DPPC/PEG40-S microbubbles, the microbubble suspension was centrifuged at 500*g* for 15 min, the subnatant was discarded, and the microbubble cake was resuspended in 3 mL of 0.9% w/v NaCl aqueous solution. This process was repeated three times, and the final suspension was resuspended in 3 mL of 0.9% w/v NaCl aqueous solution.

Poractant alfa was diluted to 5.3 mg/mL using 0.9% w/v NaCl aqueous solution up to 3 mL in an 8 mL vial. The diluted poractant alfa was then homogeneously dispersed for 20 s using a sonicator (Q125, QSonica, Newtown, CT, USA) at a power setting of 50% with the tip completely immersed in the lipid solution. The obtained solution was placed in a temperature-controlled shaker at 35 °C and 800 rpm. The sonicator tip was placed at the air–aqueous interface and sonicated for 30 s at a power setting of 90%. The headspace of the 8 mL vial was filled with air. Immediately after production, the vial containing the MB suspension was capped and placed in an ice box.

The PEG-free microbubble formulation was composed of DPPC, DPPG, and palmitic acid in 20% w/w propylene glycol in 0.9% w/v NaCl aqueous solution. This formulation was developed to exclude PEGylated lipids from the formulation. DPPG and palmitic acid replaced the role of PEGylated lipids (i.e., amphiphilic lipids that stabilize DPPC and the gas core in the aqueous environment^18^). The lipid film was first prepared. Eight mg of DPPC (Avanti Polar Lipids, USA), 8 mg of DPPG (Avanti Polar Lipids, USA), and 2 mg of palmitic acid (Sigma-Aldrich, UK) in chloroform (Sigma-Aldrich, UK) were mixed in an 8 mL glass vial. The mixture was subsequently dried using a vacuum evaporator at 30 °C for 3 h to allow for chloroform to evaporate. The obtained dry lipid film was suspended in 4 mL of 20% w/v propylene glycol in 0.9% w/v NaCl aqueous solution (i.e., the lipid suspension has a DPPC concentration of 2 mg/mL or a total lipid concentration of 4.5 mg/mL) for 1 h in a temperature-controlled shaker at 60 °C and at 800 rpm. Lipids were then homogeneously dispersed for 60 s using a sonicator (Q125, QSonica, Newtown, CT, USA) at a power setting of 50% with the tip completely immersed in the lipid solution. The lipid suspension was further diluted by 8 times using 20% w/w propylene glycol in 0.9% w/v NaCl aqueous solution to make a DPPC concentration of 0.25 mg/mL or a total lipid concentration of 0.5625 mg/mL. This is referred to as PEG-free lipid suspension.

The PEG-free lipid suspension was subsequently used to make microbubbles. The lipid suspension was first degassed, and then PFB gas was added to the headspace. CapMix (3M, UK) was used to make the microbubbles by the shaking method. The speed was set at 4,300 rpm and was shaken for 15 s. Immediately after production, the vial containing the microbubble suspension was capped and placed in ice.

For microbubble sizing and counting, 10 μL of the microbubble suspensions was put onto a Neubauer-enhanced cell counting chamber (Hausser Scientific Company, USA) under a 24 mm by 24 mm glass coverslip (VWR International, USA). A CCD camera (MicroPublisher 3.3 RTV, QImaging, Canada) and a Leica DM500 microscope (Leica Microsystems GmbH, Germany) were used to image MBs at a magnification of 40. MATLAB code that was built explicitly for microbubble sizing and counting was used (The Mathworks Inc., USA).^19^

### Creating a Physiologically Relevant Air–Aqueous Interface to Model nRDS

Lung lining fluid comprises various salts, surfactants, cells, and proteins. Out of the numerous components, salt composition, surfactants, and surfactant proteins play crucial roles in determining the surface tension of the air–aqueous interface. In this study, a physiologically relevant air–aqueous interface for nRDS was created. Gamble’s solution^20^ was used as the aqueous subphase to replicate the salt composition. Poractant alfa was used to replicate the amount of surfactant and surfactant proteins and the surface tension at the air–aqueous interface.

The quantity of poractant alfa added to the air–aqueous interface was selected based on previous studies. Stichtenoth et al. reported that the amount of phosphatidylcholine for preterm neonates born before 32 weeks of gestation could be as low as 0.04 mg/mL, and the surface tension at the initial adsorption was approximately 50 mN/m.^21^ Merrill found in a separate study that minimum surface tension was above 5 mN/m in preterm neonates with RDS and increased significantly when the percentage of SP-B/phospholipid ratio was below 1%.^22^ To create a conservative in vitro model for nRDS, 26.6 μg/mL poractant alfa (i.e., equivalent to 13.25 μg/mL phosphatidylcholine, 10 μg/mL DPPC, and 0.14 μg SP-B) was added to the subphase.

### Equilibrium and Dynamic Surface Tension Measurements

**Figure 3:**
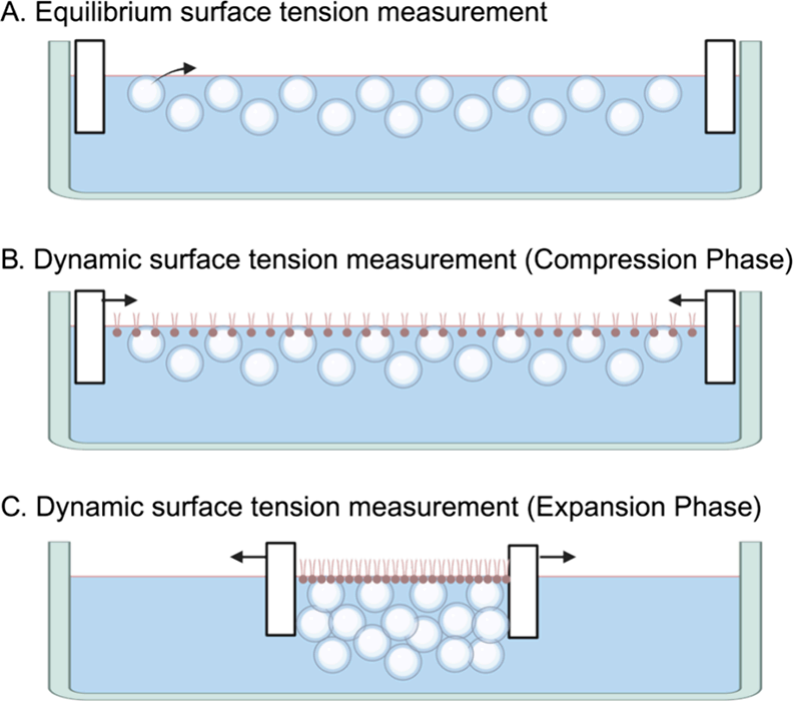
Schematic showing the method of equilibrium and dynamic surface tension measurement using the Langmuir–Blodgett trough. (A) Equilibrium surface tension measurement. Microbubble suspensions were added to the Langmuir–Blodgett trough, and the change in surface tension of the air–aqueous interface was measured. Microbubbles were found adjacent to the air–aqueous interface because of the flotation effect. The black arrow represents the transfer of surfactants from microbubble suspension to the air–aqueous interface. (B, C) Dynamic surface tension measurement. Panels (B) and (C) show the microbubbles adjacent to the surfactants on the air–aqueous interface, and the effect of microbubbles on the air–aqueous surface tension is measured for the compression phase (B) and for the expansion phase (C).

Surface tension was measured using the Wilhelmy plate method in a computer-controlled Langmuir–Blodgett instrument (MicroTrough XS, Kibron, Malminkaari, Helsinki, Finland). The tip of the plate was submerged in the subphase (i.e., aqueous solution), and the force of surface tension pulling the tip downward was measured (Figure 3).

For the clean air–aqueous interface study, the subphase was filled with 67 mL of 0.9% w/v NaCl aqueous solution. The subphase temperature was heated to 37 °C by using a temperature-controlled water bath. Samples were injected into the subphase, and the equilibrium surface tension was deemed to have been reached when fluctuations were smaller than 0.1 mN/m over a minute. The 0.1 mN/m per minute threshold is set to determine the equilibrium surface tension due to the evaporation of the aqueous subphase, causing a change in concentration. Poractant alfa reaches equilibrium surface tension within 5 min, with a rate of change faster than 0.1 mN/m per minute.^23^ DPPC exhibits two-phased adsorption, with a faster rate of surface tension reduction than 0.1 mN/m per minute.^24^

The initial area was 12,000 mm^2^. Dynamic surface tension measurements were started after the surface tension had equilibrated. The barriers on the Langmuir–Blodgett trough compress from 12,000 to 2,500 mm^2^ and vice versa for expansion. 12,000 and 2,500 mm^2^ were the maximum and the minimum surface area achievable by the Langmuir–Blodgett trough, respectively, and it facilitated the study of the microbubble behavior on the air–aqueous interface in an exaggerated expansion and compression. The compression and expansion rates were both 140 mm^2^/s, and the measurements from the second cycle are presented in the results.

For the simulated lung fluid study, the subphase was filled with 67 mL of the simulated lung fluid in a Langmuir–Blodgett Trough. The surfactants were slowly added to the air–aqueous interface directly, and the equilibrium surface tension was measured. For the dynamic surface tension, the surface area was reduced from 6,000 to 2,700 mm^2^ during the compression phase and expanded back to 6,000 mm^2^ during the expansion phase. The surface area for the dynamic expansion and compression was changed (from 12,000–2,500 mm^2^ cycle to 6,000–2,700 mm^2^ cycle) to increase the rate of each cycle per minute and lower the percentage of surface area reduction, which is closer to the physiological condition of a breathing lung.

The behavior of microbubbles on the Langmuir–Blodgett trough was also observed using a CCD camera (MicroPublisher 3.3 RTV, QImaging, Canada) and a Leica DM500 microscope (Leica Microsystems GmbH, Germany) at a magnification of 4 or/and 10.

## Results and Discussion

### Effect of Microbubbles (PEGylated) on a Clean Air–Aqueous Surface Tension

**Figure 4:**
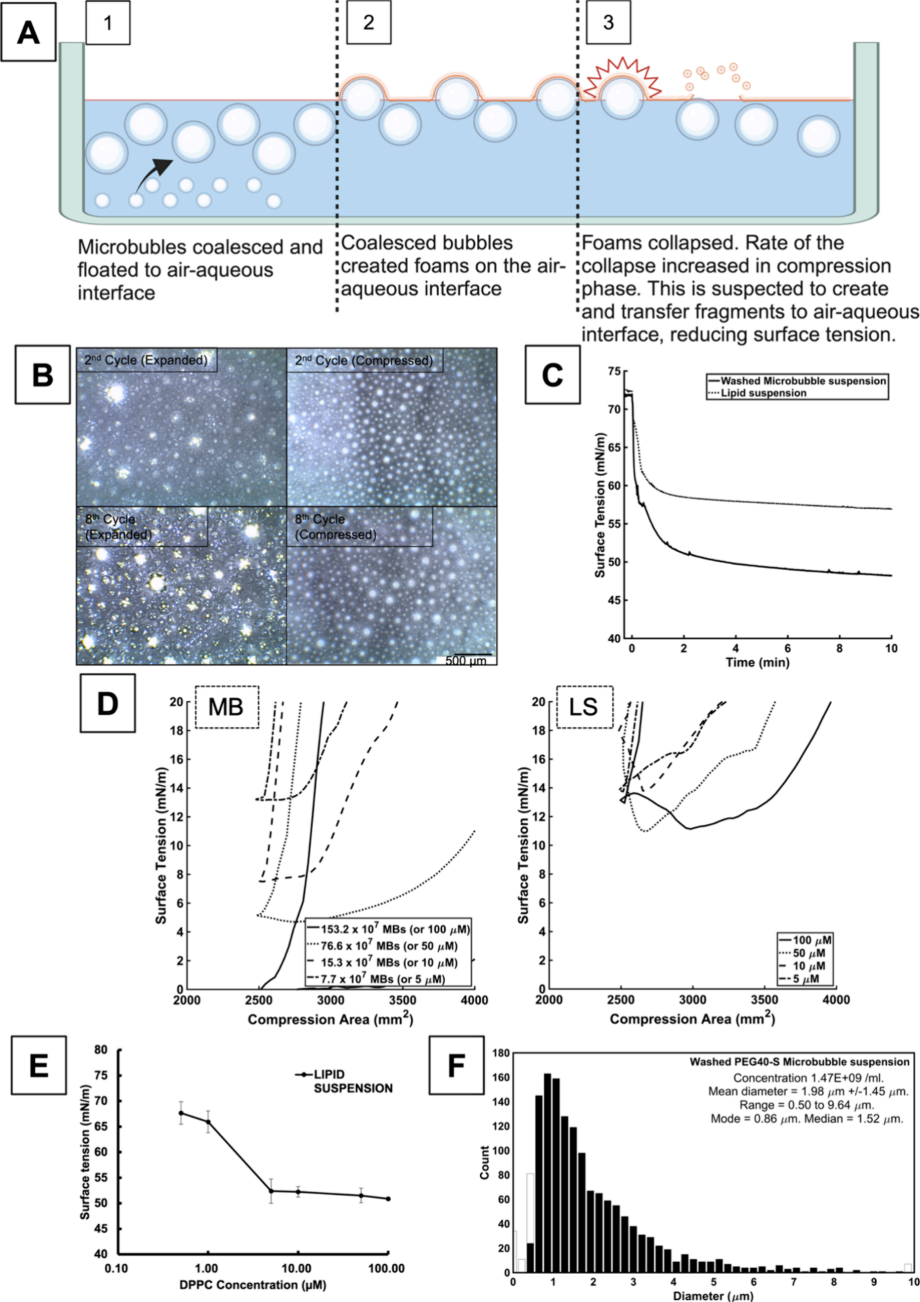
(A) Schematic that shows the behavior of PEGylated microbubbles in a clean air–aqueous interface. Microbubbles coalesced to form larger bubbles, which floated toward and rested adjacent to air–aqueous interface. Some of the bubbles became foams. Bubbles and foams ruptured. The rupture, which is known to create surfactant fragments, is suspected to transfer the surfactants onto the interface, reducing the surface tension. (B) The images were taken with a magnification objective of 4. The expanded images were taken when barriers were fully expanded (creating a surface area of 12,000 mm^2^), and the compressed images were taken when barriers were fully compressed (surface area of 2,500 mm^2^). Coalesced microbubbles are characterized by circular boundaries without a bright white color. The foams, on the other hand, do not have clear boundaries and have a bright white color on the images, which suggests light reflection on their surface. The foams were present only in the expanded phase of the compression/expansion cycle, and their numbers increased with the rounds of the cycle. Compression of the surface area destroyed the foams, thus leaving no foams at the end of the compressed phase. Foams are replenished during the expansion phase of the cycle. (C) The surface tension of the air–aqueous interface created by washed microbubbles was compared to that created by the lipid suspension. The lipid suspension and the microbubbles had the same lipid composition and concentration. The volume of suspension equivalent to 62.11 μg of DPPC was added to 67 mL of 0.9% w/v sodium chloride aqueous solution. (D) Dynamic surface tensions of DPPC/PEG40-S microbubbles (MB, left graph) and lipid suspension (LS, right graph) were measured at various concentrations of DPPC. The micromolar concentration represents the concentration of DPPC in 67 mL of 0.9% w/v NaCl aqueous subphase. The area was compressed from 12,000 to 2,500 mm^2^ and then expanded back to 12,000 mm^2^. The right line of the hysteresis represents the compression phase, and the left line (which has a steeper gradient at around 2,500 mm^2^) represents the expansion phase. The minimum surface tensions reached by microbubble and lipid suspension at 100 μM were significantly different (*n* = 3, *p* < 0.01, *t* test). The *x*-axis is partially presented from 2,000 to 4,000 mm^2^ range because it was the region of interest that showed the differences in the minimum surface tension between the microbubbles and lipid suspension. (E) Relationship between the concentration of lipid suspension and the air–aqueous surface tension (*n* = 3). The subphase is 67 mL of 0.9% w/v NaCl aqueous solution. Error bars indicate the standard deviation. Increasing the concentration of lipid suspension reduced the surface tension down to 50.0 mN/m where the plateau reached 10 μM. This suggested that the critical micelle concentration (CMC), or the concentration above which the air–aqueous surface tension no longer reduces, is achieved at around 10 μM. (F) Size distribution of washed PEGylated microbubbles as measured using light microscopy (three batches of samples were measured with 20 images for each sample). The microbubbles used in this study had a concentration of 1.47 × 10^9^ microbubbles/ml and a median diameter of 1.52 μm.

The behavior of microbubbles on a clean air–aqueous interface and its subsequent effect on the surface tension was studied. PEGylated microbubbles were washed to remove as many of the other macromolecular structures (e.g., liposomes, micelles, and potentially multilamellar structures) as possible from the microbubble suspension. The resulting microbubble population is shown in Figure 4F.

The change in surface tension of a clean air–aqueous interface by washed microbubbles was compared to that produced by a lipid suspension with the same composition. Figure 4C shows that the microbubbles achieved a lower air–aqueous surface tension than the lipid suspension (48.2 vs 57.0 mN/m, respectively), and the rate of the decrease was faster with the microbubbles. This is likely due to flotation. A large proportion of the microbubbles have sufficiently large size (>1.0 μm) that buoyancy would have dominated their movement^21^ (Figure 4F). The flotation of microbubbles created a greater surfactant concentration gradient between the air–aqueous interface and the adjacent aqueous subphase than that the lipid suspension and thus increased the rate of reduction in the surface tension.

It was found that there is a mechanism(s) other than the increased concentration gradient that contributed to the faster reduction in surface tension. Critical micelle concentration (CMC) for DPPC/PEG40-S lipid suspension is obtained by looking at Figure 4E, and it was found that the air–aqueous surface tension at CMC (50.0 mN/m) was higher than the surface tension achieved by the washed microbubbles (48.2 mN/m). This suggested that microbubbles have a different mechanism(s) of transferring the surfactants (i.e., DPPC and PEG-40S) to the air–aqueous interface than that of a lipid suspension.

One possible mechanism is foam or bubble rupture (as described in the schematic in Figure 4A). When observed under the microscope, microbubbles were found to coalesce into large bubbles (in hundreds of micrometers size range), and the large bubbles floated to the air–aqueous interface (Figure 4B). The behavior of the large bubbles on the air–aqueous interface was previously studied,^25,26^ and found that the large bubbles collide with the interface, leading to either a bubble rupture or the formation of foam (which then ruptures with time). In a separate study, a bubble rupture has been shown to deposit bubble film material directly onto an air–water interface.^26^ Similarly, foam film rupture creates regions of densely packed surfactants.^27^ This study has found that such foam/bubble rupture of the lipids can increase surfactant adsorption on the air–aqueous interface and lead to a reduction in surface tension below the CMC.

The behavior of microbubbles during compression and expansion and their effect on the dynamic surface tension was studied (Figure 4D). Dynamic surface tension measurements with the DPPC/PEG40-S unwashed microbubble suspension were compared to those with the corresponding DPPC/PEG40-S lipid suspension. The difference in the dynamic surface tension between the two formulations was striking below the air–aqueous surface tension of 20 mN/m. A DPPC monolayer on an air–aqueous interface is known to enter a collapsing state at around 20 mN/m of surface tension.^28^ The collapsing state is inherently unstable and results in the desorption of DPPC into the subphase in the form of vesicles.^29^ The rate of collapse, thus, limits the minimum surface tension that can be reached during compression.^28^ The microbubble suspension with the final subphase DPPC concentration of 100 μM achieved air–aqueous surface tension near 0 mN/m, while the lipid suspension, with the same concentration, achieved a minimum surface tension of around 10 mN/m.

Foam and bubble rupture could again explain how microbubbles achieved a lower air–aqueous surface tension than the lipid suspension during compression (Figure 4B).

### Microbubbles Improve the Surface Activity of Poractant Alfa in an Air–Saline Interface

**Figure 5:**
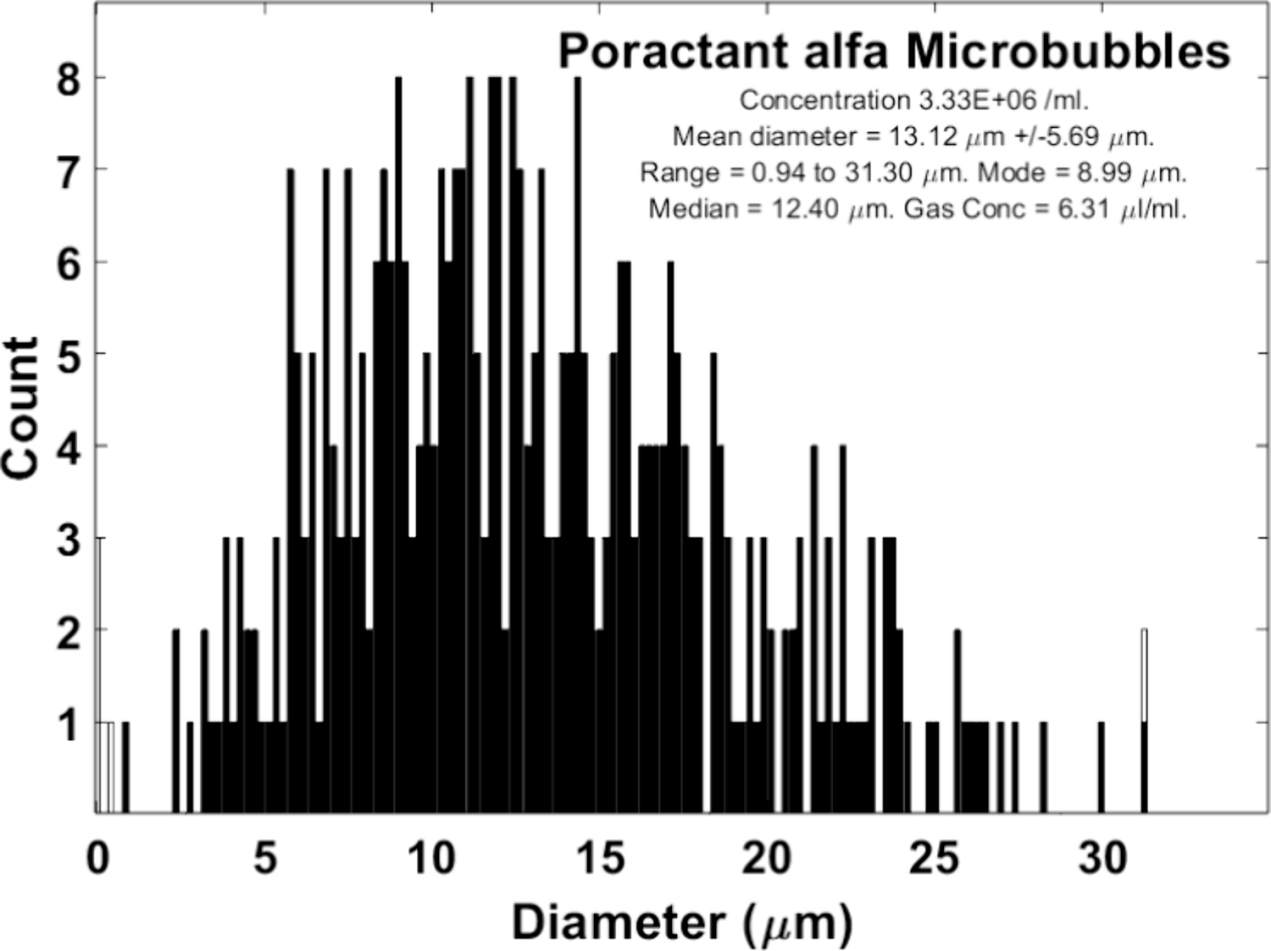
Size characteristics of the poractant alfa microbubbles (PaMBs) are presented (three batches of samples were measured with 20 images for each sample).

Microbubbles were manufactured with diluted poractant alfa and an air gas core (PaMBs), and had a lower concentration (6.66 × 10^6^ /ml), larger diameter (i.e., mean diameter was 13.1 μm), and broader size distribution (i.e., the range of diameter was 0.9 to 31.3 μm) than the PEGylated microbubbles (Figure 5).

**Figure 6:**
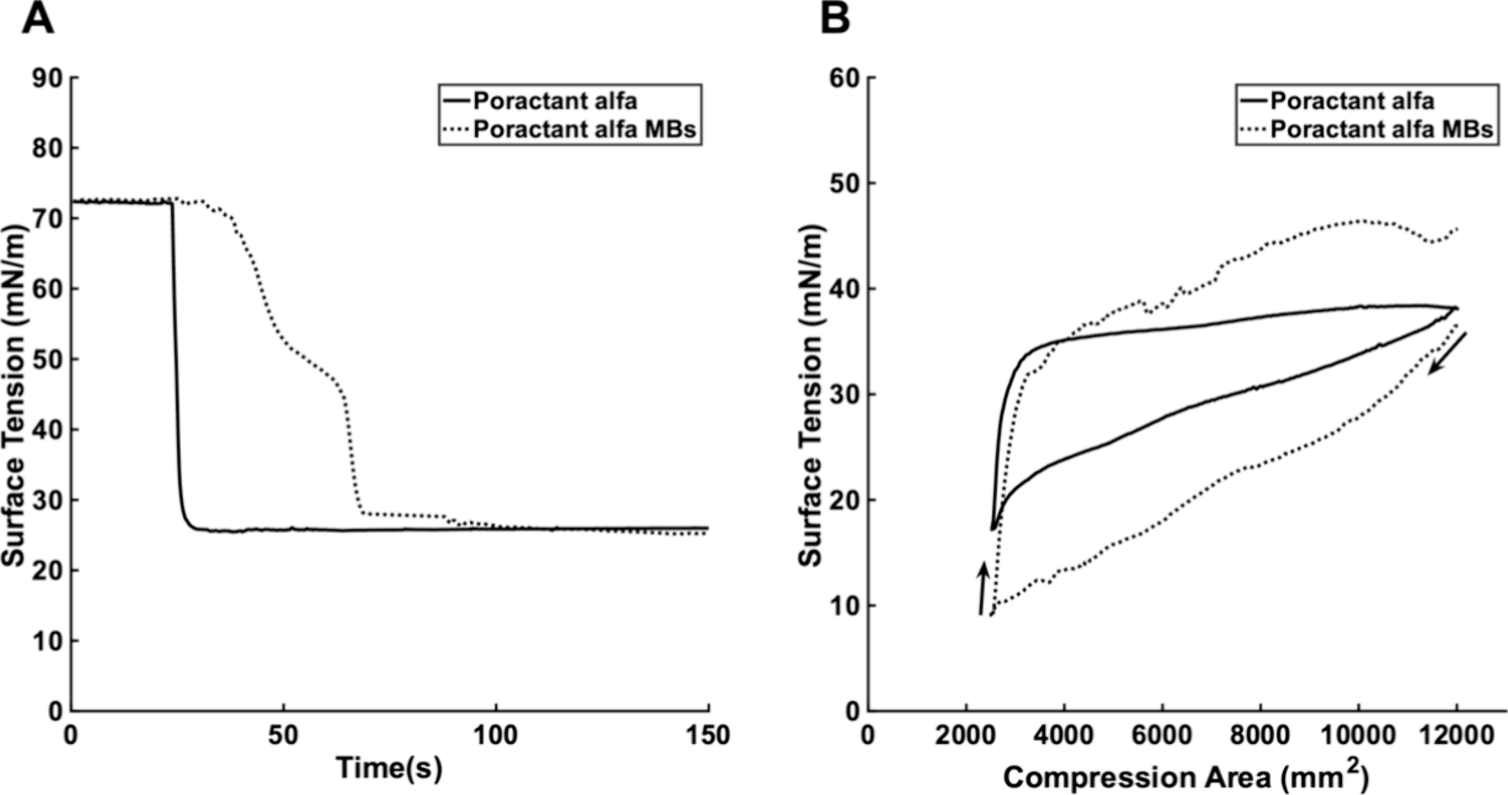
(A) The effect of poractant alfa or PaMBs on equilibrium surface tension was measured at a DPPC concentration of 195.75 mg/mL (equivalent to 10 μM of DPPC). (B) The effect of poractant alfa or PaMBs on dynamic surface tension was measured at a DPPC concentration of 195.75 μg/mL. The subphase was 0.9% w/v NaCl aqueous solution. The area was compressed from 12,000 to 2,500 mm^2^ and then expanded back to 12,000 mm^2^. The black arrows indicate the compression/expansion phase. The minimum surface tension achieved by poractant alfa was significantly different from that of the corresponding microbubbles (*n* = 3, *p* < 0.01, *t* test).

PaMBs achieved equilibrium surface tension similar to that of poractant alfa (Figure 6A), but the dynamic surface tension during the compression and expansion cycle dramatically differed between the PaMBs and poractant alfa (Figure 6B). During the compression phase, PaMBs achieved lower air–aqueous surface tension than poractant alfa (8.91 vs 17.18 mN/m). PaMBs, however, had unfavorable characteristics for use in nebulized lung surfactant therapy. They could not be nebulized using a vibrating mesh nebulizer (e.g., e.g., AeroNeb by Aerogen) because the majority of PaMBs were larger than 3 μm in diameter (mesh holes have diameters below 3 μm). In a separate preliminary study, it was confirmed that microbubbles did not survive nebulization (the study protocol can be found in ref (13)). Additionally, PaMBs had poor stability and disappeared within 15 min at 37 °C. This partly explained the increase in the air–aqueous surface tension at the end of the compression/expansion cycle (Figure 6B). Microbubbles with smaller diameters, higher concentrations, and better stability were required for nebulized lung surfactant therapy. Poractant alfa, an animal-derived product, also has other disadvantages, such as a risk of infection, high cost, low availability, and difficulty in manufacturing.

### Effect of PEG-Free Microbubbles on the NRDS-Relevant Model Air–Aqueous Interfacial Surface Tension

**Figure 7:**
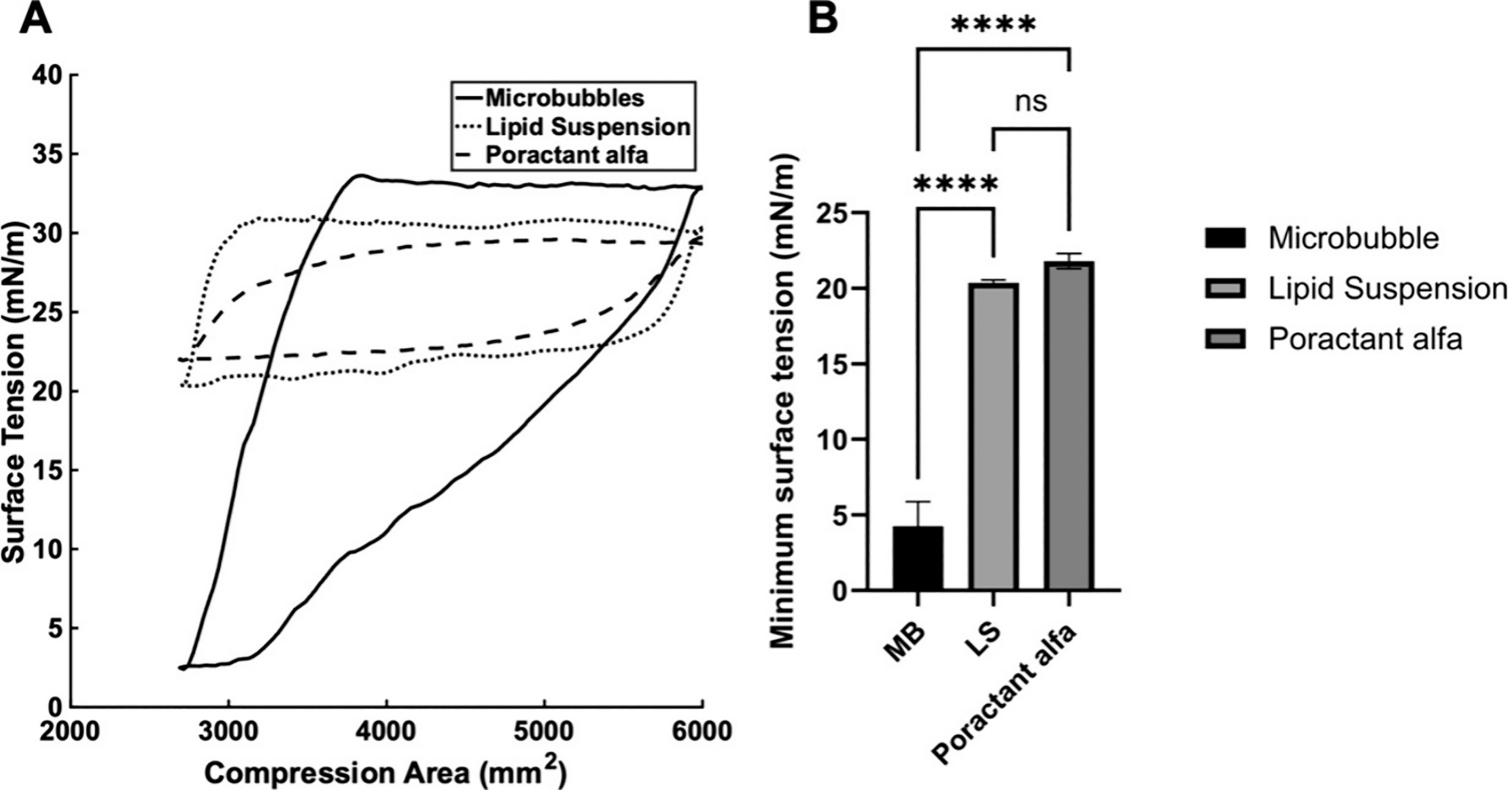
(A) The effect of poractant alfa, a PEG-free lipid suspension, or PEG-free microbubbles on dynamic surface tension was measured. Volume equivalent to 670 μg of DPPC (or an additional 10 μg/mL in the subphase) was added to the subphase. The subphase was an nRDS-relevant air–aqueous solution. The area was compressed from 6,000 to 2,700 mm^2^ and then expanded back to 6,000 mm^2^. (B) The minimum surface tensions of the air–aqueous interface achieved by microbubbles (MB), lipid suspension (LS), and poractant alfa were compared (*n* = 3, ANOVA). **** represents *p* ≤ 0.0001.

The effects of the three groups (PEG-free microbubbles, PEG-free lipid suspension, and poractant alfa) on the dynamic surface tension of the model air–aqueous interface were studied (Figure 7). Microbubbles reduced surface tension below 5 mN/m (i.e., 1.6 mN/m), whereas the lipid suspension and poractant alfa produced minimum surface tensions of 20.3 and 21.8 mN/m, respectively, at the same DPPC concentration (in fact, the poractant alfa had a higher total lipid concentration). This suggested that microbubbles interact differently with the phospholipid monolayer from the other structures present in lipid suspension (e.g., liposomes and micelles).^30^

**Figure 8:**
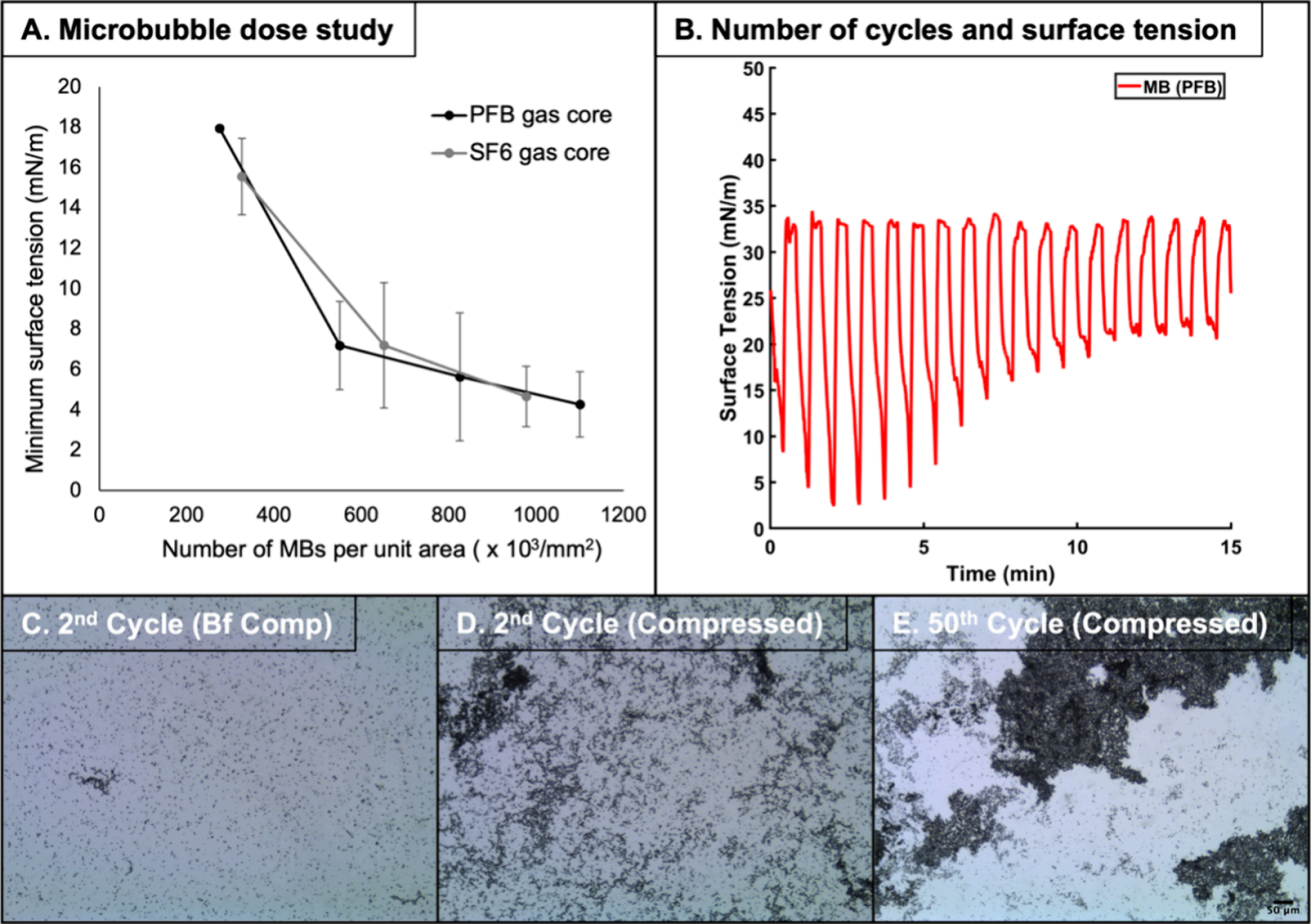
(A) The number of microbubbles determined the minimum surface tension reached during the compression phase of the cycle (*n* = 3). The compression area was reduced from 6,000 to 2,700 mm^2^. (B) The change in the surface tension with cycles of compression and expansion is shown. The area was again reduced from 6,000 to 2,700 mm^2^ and expanded vice versa. The experiment was repeated three times. C-E show images of microbubbles during compression and expansion cycles. The magnification is 10. (C) is an image of microbubbles on the air–aqueous interface taken at the end of 1st expansion phase and just before the 2nd compression. Panel (D) is taken after the 2nd compression. Panel (E) is taken after 50 cycles of compression and expansion cycles and taken at the compressed state. Clustering of microbubbles is speculated to hinder microbubbles’ ability to reduce surface tension during the compression phase because the microbubbles cover less surface area on the air–aqueous interface and thus exert less of their effect on the phospholipid monolayer during the compression phase.

The mechanism(s) by which the microbubbles reduced the air–aqueous surface tension in the nRDS-model interface appeared to be related to the clusters of microbubbles below the air–aqueous interface. The microbubbles were seen to be floating near the air–aqueous interface and to form clusters upon compression of the air–aqueous surface area. Each compression cycle created new or increased the size of clusters of microbubbles near the interface. The cluster(s) of microbubbles at the end of the 50th cycle was dramatically larger than the clusters in the second compression cycle (Figure 8C–E).

The following pieces of evidence suggest that the microbubbles are instrumental for the low minimum surface tension and are being used up to reduce the minimum surface tension. They, therefore, indirectly substantiated that the microbubble-phospholipid monolayer interaction exists. The number of microbubbles directly determined the minimum surface tension reached during the compression phase (Figure 8A). This suggested that the microbubbles are instrumental in low air–aqueous interfacial tension. Compression and expansion cycles reduced the number of microbubbles. The number of microbubbles per mm^2^ of the air–aqueous interface was reduced from 2.96 × 10^8^ MBs/mm^2^ at the end of the second cycle to 7.18 × 10^7^ MBs/mm^2^ at the end of the 50th cycle (Figure 8C–E). The number of microbubbles was counted using the MATLAB code built explicitly for microbubble sizing and counting (The Mathworks Inc., USA).^18^ Reduction in the number of microbubbles led to an increase in the minimum surface tension reached during the compression phase of the cycle after the first few (Figure 8B). This indirectly proves that the microbubbles are transferring phospholipids onto the air–aqueous interface, but the exact mechanism of the transfer has yet to be found and needs further investigation.

### Clinical Considerations for Microbubbles for Nebulized Lung Surfactant Therapy

A minimum surface tension below 5 mN/m is the in vitro marker for the efficacy of surfactants.^7,21,22,31^ The minimum surface tension is instrumental in predicting the likelihood of airway or alveoli collapse because low surface tension prevents overextension of the lung airways, and its recoil effect leads to the collapse. The collapsed airway is also more likely to reopen with low surface tension on the air–aqueous interface. The minimum surface tension of the air–aqueous interface achieved by microbubbles was comparable to that of healthy neonates. Air–aqueous surface tension achieved by the lung aspirates from healthy neonates was below 5 mN/m, and ranged from 0.4 to 4.7 mN/m,^31^ and microbubbles achieved the minimum surface tension of 4.2 mN/m during the compression phase of the cycle. This is strikingly low compared to air–aqueous surface tension in preterm neonates with nRDS^21,22^ whose minimum surface tension ranges from 26.8 to 44.4 mN/m. With the same DPPC concentration, microbubbles were able to achieve substantially lower surface tension during compression than poractant alfa (4.2 vs 21.8 mN/m).

The lack of efficacy reported in a recently terminated clinical Phase 2 trial of nebulized lung surfactant therapy in preterm neonates was attributed to an insufficient amount of surfactant entering the lungs.^11^ Poor lung delivery is an inherent problem for pulmonary administration, and this problem is accentuated in preterm neonates because of their narrow airways. Lung deposition of inhaled aerosols in 4-week-old babies was found to be 4.5% at 2 L/min flow rate and 2% at 3 L/min.^9^ Given that preterm babies have narrower airways and 8 L/min is the flow rate used for nRDS, it is expected that lung deposition would be considerably lower than 2% in preterm neonates.

Preterm neonates with less than 32 weeks gestation have yet to form alveolar sacs, and thus, the surface area in their lungs is much smaller than in healthy neonates. It is estimated that the lung surface area in these extreme preterm neonates is around 637.7 cm^2^ (or 63770 mm^2^).^32^ In the present study, it was found that 800,000 MBs/mm^2^ were required to achieve a minimum surface tension below 5 mN/m, and therefore, approximately 51 × 10^9^ microbubbles would need to be delivered to the lungs. Assuming that lung deposition is 5% for microbubble-laden aerosols, 255 mL of microbubble suspension would be required, and it would take 1020 min (or 17 h) to administer a sufficient dose (assuming a concentration of microbubbles is 4 × 10^9^ MBs/mL and an output rate of the vibrating mesh nebulizer of 0.25 mL/min). The current formulation is, therefore, not likely to be suitable for clinical use. Potential means of overcoming these challenges include: improving the surface activity of the microbubbles, so that fewer are required to reduce the surface area during compression/expansion cycles; increasing the microbubble concentration to reduce the volume of microbubble suspension required to be nebulized; and/or improving lung deposition efficiency, again, reducing the volume of microbubble suspension required to be nebulized.

## Conclusions

This study investigated whether microbubbles could be used to improve surfactant delivery to an air–water interface and hence potentially offer a more efficient treatment method for nRDS. It was found that phospholipid-coated microbubbles did improve air–water interface adsorption of phospholipids compared with the equivalent liquid suspension and achieved a surface tension close to zero at lower molecular concentrations during compression of air–aqueous surface area. Further study is required to (1) understand the mechanism(s) by which microbubbles reduce air–aqueous surface tension during compression and (2) to confirm improvement in lung compliance or/and oxygenation with microbubble surfactant therapy in vivo.
